# Strategy for initial en bloc resection of a giant mediastinal solitary fibrous tumor: Judicious usage of cardiopulmonary bypass

**DOI:** 10.1111/1759-7714.13477

**Published:** 2020-05-07

**Authors:** Lei Chen, Yonghua Sang, Zhiwei Zhang, Wentao Yang, Yongbing Chen

**Affiliations:** ^1^ Cardiothoracic Surgery The Second Affiliated Hospital of Soochow University Suzhou China

**Keywords:** cardiopulmonary bypass, en bloc resection, mediastinal solitary fibrous tumor

## Abstract

A solitary fibrous tumor (SFT) is a rare mediastinal neoplasm associated with a high recurrence rate. Total excision on initial surgery is an established indicator of a positive outcome. Here, we report the case of a 52‐year‐old man who was admitted to our hospital with symptoms of cough, chest pain, and dyspnea for two months. Chest computed tomography (CT) scan revealed a middle mediastinal mass which infiltrated adjacent vital structures, and surgery was performed with the assistance of cardiopulmonary bypass (CPB) and median sternotomy. The mass was completely removed and histopathology confirmed the presence of a mesenchymal tumor. The patient had an uneventful recovery without any perioperative symptoms, hoarseness, or dysfunction of the diaphragm. Sixty‐nine months after surgery, a CT scan confirmed that the patient remained disease‐free without necessitating the introduction of chemotherapy or radiotherapy. Here, to the best of our knowledge, we report the first case of a giant invasive mediastinal SFT that was completely resected during initial surgery under CPB with a remarkable outcome.

## Introduction

A solitary fibrous tumor (SFT) is a rare mediastinal neoplasm associated with a high recurrence rate.^1^ Complete excision at initial surgery is an established indicator of a positive outcome.^2^ Several CPB (cardiopulmonary bypass)‐induced complications may occur, such as bleeding related to systemic heparinization. Therefore, resection of the SFT is routinely performed by median sternotomy, or thoracotomy without the assistance of CPB. However, routine surgical procedures may actually contribute to the high rate of local recurrence and consequently a poor outcome. Here, to the best of our knowledge, we report the first case of a giant invasive mediastinal SFT infiltrating neighboring vital structures that was completely resected during initial surgery under the assistance of CPB with a remarkable outcome.

## Case report

**Figure 1:**
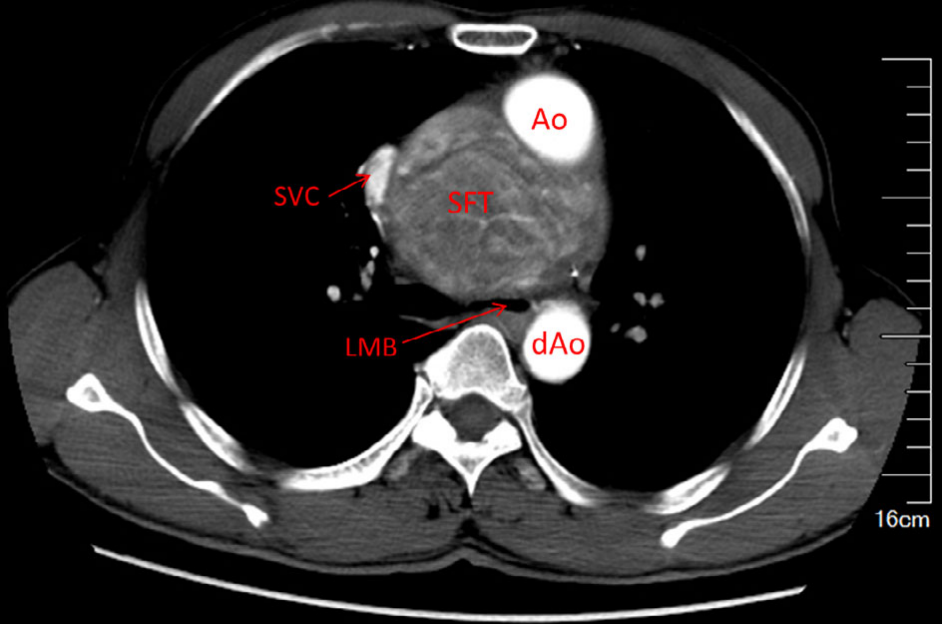
Thoracic computed tomography (CT) scan showing a giant mediastinal mass infiltrating adjacent vital structures.

In 2014, a 52‐year‐old man with symptoms of cough, chest pain, and dyspnea for two months was admitted to our hospital. The patient reported no exposure to asbestos, radon gas, or other occupational toxicants. Chest computed tomography (CT) scan revealed an 8.5 × 9.5 cm middle mediastinal mass which was infiltrating adjacent vital structures (Fig 1). Due to the undeniable challenges of surgical resection, he had previously been refused care by several hospitals.

**Figure 2:**
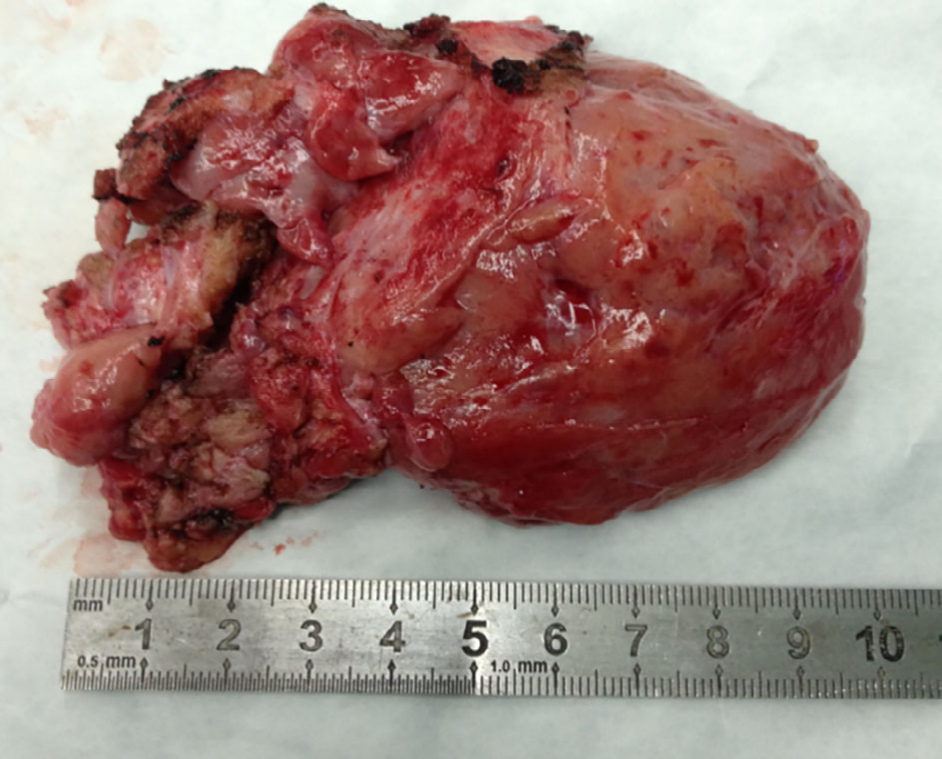
En bloc resection of the solitary fibrous tumor (SFT) with the assistance of CPB at the initial surgery.

**Figure 3:**
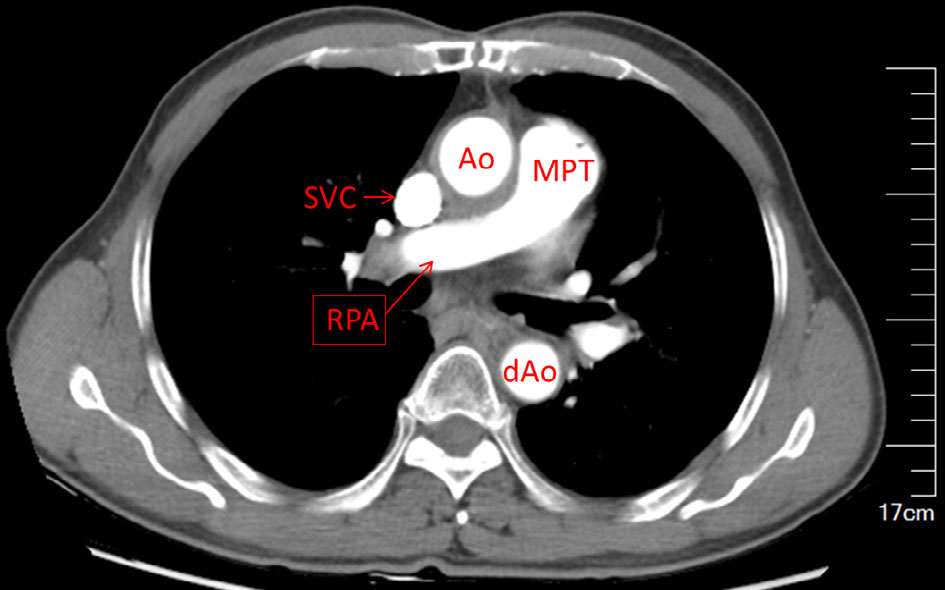
Thoracic CT scan showing no recurrence at 69 months after surgery.

The initial surgery was performed with the assistance of cardiopulmonary bypass (CPB) and median sternotomy. A firm and hypervascular mass was found encircling the right pulmonary artery (RPA) with compression and anterolateral displacement of the superior vena cava (SVC), as well as compression of the ascending aorta (AAO) and left main bronchus (LMB), but no invasion of the phrenic nerve or recurrent laryngeal nerve. The mass was completely and meticulously dissected (Fig 2). Because of dense adherence to the SVC and RPA, the side walls of the SVC and RPA were repaired with 5–0 polypropylene sutures without arteriovenous grafting. However, there was a significant decrease in CVP from 20 to 8 cm H_2_O, while other vital signs remained stable during the procedure. Histopathological analysis confirmed the presence of a mesenchymal tumor. Results of immunohistochemical staining were positive for CD99, CD34, Bcl‐2, and vimentin, but negative for S‐100, desmin, and SMA, confirming the mass as an invasive SFT. Recovery was uneventful without any perioperative symptoms, hoarseness, or dysfunction of the diaphragm. At 69 months after surgery, a CT scan confirmed that the patient remained disease‐free without necessitating the introduction of chemotherapy or radiotherapy (Fig 3).

## Discussion

After reviewing the scarce SFT literature, there are four types of combined therapeutic strategies which are summarized as follows^1^, ^3^, ^4^, ^5^, ^6^, ^7^: (i) Neoadjuvant chemoradiotherapy plus surgery; (ii) surgery alone; (iii) surgery plus adjuvant chemoradiotherapy; or (iv) chemoradiotherapy alone. There has been no systematic evaluation of therapeutic effect because reports are rare.^8^ As there is no relevant criteria for therapeutic strategies, clinicians can select any strategy according to personal experience, no matter the nature of the tumors. As the effectiveness has not been proved, the role of chemoradiotherapy in the treatment of SFT remains controversial.

Several complications may occur attributed to CPB, with bleeding related to systemic heparinization as the major concern.^3^, ^9^ Consequently, thoracic surgeons are inclined to perform the surgical procedure by median sternotomy or thoracotomy without CPB. Under these circumstances, thoracic surgeons are unable to rule out the possibility of a further resection by CPB due to local recurrence after right lateral thoracotomy.^3^ Therefore, it is reasonable to conclude that routine surgical procedures may lead to the high rate of local recurrence and poor outcomes.

We chose initial en bloc resection by CPB because incomplete resection is associated with poor prognosis for such a giant invasive mediastinal SFT. The following considerations confirmed our decision: (i) compression of the AAO, SVC and RPA existed, which may result in hemodynamic disorder, especially acute right ventricular dysfunction secondary to the sudden relief of the prolonged obstruction of the SVC; and (ii) given that the LMB was compressed, there would be a risk of hypoxia during surgery, as previously reported.^10^ In an en bloc resection, CPB is applied to ensure hemodynamic and respiratory stability.

The recurrence rate of benign classification is only 2% after surgical excision. Approximately half of malignant classification often develop recurrences and metastases.^1^ SFT have been reported to metastasize usually via the hematogenous route,^11^, ^12^ and recurrences and metastases have previously indicated poor outcomes.^13^ Chemoradiotherapy was not used even though the mass was an invasive SFT because a successful initial en bloc resection had been made during the operation with the assistance of CPB. During the 69 months follow‐up, our patient remained disease‐free, even though the immunohistochemical staining suggested a malignant SFT. We are of the opinion that the favorable prognosis was due to initial en bloc resection with the vital assistance of CPB.

The vascular supply to the mass in the present case originated from the thoracic aorta. To date, there has been only one case report on the vascular supply of a mediastinal SFT, which was from the coronary arteries, and no other reports have described the vascular supply.^14^ We have reported the precise positioning of the vascular supply to avoid unpredictable bleeding, which may inspire clinicians to explore new interventional therapies that may be more beneficial than chemoradiotherapy in the future.

## Disclosure

The authors declare no conflicts of interest.
